# A Quasi-Experimental Study Analyzing the Effectiveness of Portable High-Efficiency Particulate Absorption Filters in Preventing Infections in Hematology Patients during Construction

**DOI:** 10.4274/tjh.2014.0010

**Published:** 2016-02-17

**Authors:** Mehmet Özen, Gülden Yılmaz, Belgin Coşkun, Pervin Topçuoğlu, Bengi Öztürk, Mehmet Gündüz, Erden Atilla, Önder Arslan, Muhit Özcan, Taner Demirer, Osman İlhan, Nahide Konuk, İsmail Balık, Günhan Gürman, Hamdi Akan

**Affiliations:** 1 Ankara University Faculty of Medicine, Department of Hematology, Ankara, Turkey; 2 Ankara University Faculty of Medicine, Department of Infectious Diseases, Ankara, Turkey; 3 Ankara University Faculty of Medicine, Department of Internal Medicine, Ankara, Turkey

**Keywords:** HEPA filter, Infection, invasive fungal infection

## Abstract

**Objective:**

The increased risk of infection for patients caused by construction and renovation near hematology inpatient clinics is a major concern. The use of high-efficiency particulate absorption (HEPA) filters can reduce the risk of infection. However, there is no standard protocol indicating the use of HEPA filters for patients with hematological malignancies, except for those who have undergone allogeneic hematopoietic stem cell transplantation. This quasi-experimental study was designed to measure the efficacy of HEPA filters in preventing infections during construction.

**Materials and Methods:**

Portable HEPA filters were placed in the rooms of patients undergoing treatment for hematological malignancies because of large-scale construction taking place near the hematology clinic. The rates of infection during the 6 months before and after the installation of the portable HEPA filters were compared. A total of 413 patients were treated during this 1-year period.

**Results:**

There were no significant differences in the antifungal prophylaxis and treatment regimens between the groups. The rates of infections, clinically documented infections, and invasive fungal infections decreased in all of the patients following the installation of the HEPA filters. When analyzed separately, the rates of invasive fungal infections were similar before and after the installation of HEPA filters in patients who had no neutropenia or long neutropenia duration. HEPA filters were significantly protective against infection when installed in the rooms of patients with acute lymphocytic leukemia, patients who were undergoing consolidation treatment, and patients who were neutropenic for 1-14 days.

**Conclusion:**

Despite the advent of construction and the summer season, during which environmental Aspergillus contamination is more prevalent, no patient or patient subgroup experienced an increase in fungal infections following the installation of HEPA filters. The protective effect of HEPA filters against infection was more pronounced in patients with acute lymphocytic leukemia, patients undergoing consolidation therapy, and patients with moderate neutropenia.

## Abstract (tr)

**Amaç:**

Hematoloji yatan hasta kliniklerinin yakınındaki inşaat ve tamiratların neden olduğu, hastalardaki artmış enfeksiyon riski büyük bir endişe kaynağıdır. Yüksek etkinlikte partikül emici (YEPE) filtrelerin kullanımı enfeksiyon riskini azaltabilir. Bununla birlikte allojenik hematopoetik kök hücre nakli yapılanlar dışında, YEPE filtrelerin hematolojik malignite hastalarında kullanımı konusunda standart bir öneri yoktur. Bu öncesi sonrası çalışma inşaat esnasında enfeksiyonların engellenmesinde YEPE filtrelerin etkinliğini ölçmek için kurgulandı.

**Gereç ve Yöntemler:**

Hematoloji kliniğinin yanında meydana gelen geniş çaplı bir inşaat nedeniyle hematolojik tümörlerin tedavisinin yapıldığı hasta odalarına portabl YEPE filtreler yerleştirildi. Portabl YEPE filtrelerin yerleştirilmesinden önceki ve sonraki 6 aydaki enfeksiyon oranları karşılaştırıldı. Bu 1 yıllık dönemde toplam 413 hasta tedavi edildi.

**Bulgular:**

Gruplar arasında antifungal profilaksi ve tedavi rejimleri açısından farklılık yoktu. YEPE filtrelerin yerleştirilmesini takiben tüm hastalarda enfeksiyon, klinik gösterilmiş enfeksiyon ve invaziv fungal enfeksiyon oranları azaldı. Ayrı ayrı analiz edildiğinde ise YEPE filtre yerleştirilmesi sonrası ve öncesi invaziv fungal enfeksiyon oranları nötropenisi olmayan veya uzun nötropeni süresi olan hastalarda benzerdi. Akut lenfoblastik lösemi hastalarının, konsolidasyon tedavisi alan hastaların ve 1 ile 14 gün arasında nötropenik kalan hastaların odalarına yerleştirilen YEPE filtreler enfeksiyonlara karşı belirgin bir şekilde koruyucuydu.

**Sonuç:**

Çevresel Aspergillus kontaminasyonunun daha sık olduğu yaz sezonuna ve ortaya çıkan inşaata rağmen, YEPE filtrelerin yerleştirilmesi sonrası dönemde ne hastalarda ne de hasta alt gruplarında fungal enfeksiyonlarda artış gözlenmedi. YEPE filtrelerin enfeksiyonlara karşı koruyucu etkisi akut lenfoblastik lösemi hastalarında, konsolidasyon tedavisi alan hastalarda ve orta derecede nötropenisi olanlarda daha belirgindi.

## INTRODUCTION

Infectious diseases are the most common cause of mortality and morbidity in hematology inpatient clinics. The use of high-efficiency particulate absorption (HEPA) filters in bone marrow transplantation units reduces the rates of infection and transplant-related mortality in allogeneic hematopoietic stem cell transplantation (AlloHSCT) recipients [1]. The use of HEPA systems is recommended because of the high infection rates in these units [2]. Although the rates of infection are high in all neutropenic patients [3], there are no recommendations regarding the use of HEPA filters to prevent infections in non-AlloHSCT hematologic patients.

Construction near hospitals is an important contributing factor in the development of invasive fungal infections (IFIs) in patients due to environmental fungal contamination, and HEPA filters are effective in preventing IFIs [4,5,6]. The use of HEPA filters can also prevent bacterial infections [7,8,9]. To our knowledge, ours is the first study to compare the ability of HEPA filters to prevent infections in various patient groups.

## MATERIALS AND METHODS

Demolition and construction occurring near a 6-story hospital located 10 m from the hematology ward at our university provided us with the opportunity to conduct a non-randomized retrospective quasi-experimental study to evaluate the ability of HEPA filters to prevent infections in patients being treated for hematologic malignancies during the construction. All of the patients in the hematopoietic stem cell transplantation unit were excluded from the study because that unit already had HEPA filters installed. Portable H14-type HEPA filters (99.9995% effective; Uvion Air Aseptizör, Teknomar, Turkey) were installed in all the patients’ rooms on 5 May 2011.

We compared the infection rates in the 6-month periods before and after the installation of the HEPA filters to evaluate whether the filters prevented infections. A total of 413 patients were treated in our hematology ward during this 1-year period. All patients were admitted to private rooms, and preventative measures against infection were taken with all patients. The 210 patients treated between 5 November 2010 and 4 May 2011 served as the control group and the 203 patients treated between 5 May 2011 and 26 October 2011 served as the intervention group. The patients in the control group were housed in rooms without HEPA filters, and the patients in the intervention group were housed in rooms with HEPA filters. We excluded patients from the study if they acquired IFIs in other wards prior to being admitted to our inpatient hematology department.

We also randomly measured the level of airborne particulates in patients’ rooms to evaluate HEPA filter efficiency. The levels of particulates in the patients’ rooms were within acceptable limits.

Data were assembled from patients’ files, digital records, and records of infection from the control team.

### Definitions of Infections

Infections were classified as microbiologically documented infections, clinically documented infections, and fevers of unknown origin (FUOs).

Microbiologically documented infections were defined microbiologically in cultures either as bloodstream infections or infected foci [10,11].

Clinically documented infections in patients were defined by the presence of clinical signs of infections in the absence of positive cultures for pathogenic microorganisms [10,11].

FUOs were defined as isolated fevers with no clinical or microbiological signs of infection [10,11].

IFIs were defined according to EORTC/MSG (European Organization for Research and Treatment of Cancer/Mycoses Study Group) criteria [12]. Although candidemia results were given, Candida-associated yeast infections were not considered as IFIs in this study because HEPA filters are not effective in preventing yeast infections [13]. Therefore, in our study, all cases of IFIs were mold-related. Although severe neutropenia is classically defined as neutropenia persisting for more than 7 or 10 days, many experts extend this to 14 days for IFIs [2,14]. Thus, we defined severe neutropenia as neutropenia that lasted for more than 14 days for IFIs.

### Statistical Analysis

Numeric variables are given as medians or mean and range. The non-parametric Mann-Whitney U test was used to compare nominal variables. The categorized variables were compared using the chi-square or Fisher exact test. Data were analyzed using SPSS 16.0 for Windows and p-values of less than 0.05 were considered to be significant.

## RESULTS

The control and intervention groups were similar in sex distribution, underlying hematological disease, history of fungal infections, presence of central catheter, granulocyte colony-stimulating factor usage, minimum albumin levels, and severity of neutropenia (Table 1). However, patients in the intervention group tended to have a higher mean age (p=0.053).

Mean hospitalization durations were longer in the control group than in the intervention group at 20 days and 15 days, respectively (p<0.05) (Table 2). The intervention group had lower incidences of IFIs, clinically documented infections, clinically documented pneumonia, and overall infections than the control group (Table 2). The rates of FUOs, all pneumonias, bacterial pneumonias, fungal infections, probable IFIs, possible IFIs, microbiologically documented infections, gram-positive and gram-negative bacterial infections, candidemia, and infection-related mortality were similar between the groups (Table 2).

The most common bacterial infections were Streptococcus in 8 patients, Escherichia coli in 6 patients, Pseudomonas in 4 patients, Staphylococcus in 4 patients, Klebsiella in 3 patients, and Salmonella, Pneumococcus, and Acinetobacter baumannii in 1 patient each in the control group, and Streptococcus in 4 patients, E. coli in 11 patents, Pseudomonas in 2 patients, Klebsiella in 4 patients, Pneumococcus in 2 patients, and Enterococcus and Staphylococcus in 1 patient each in the intervention group. HEPA filters seemed to be effective in preventing IFIs in all neutropenic patients during construction. Careful evaluation of the data revealed that HEPA filters were more effective in preventing infections in particular subgroups of hematology patients during construction. When the subgroups were analyzed separately, the IFI-preventive effect of HEPA filters was most marked in acute lymphoid leukemia patients, especially during consolidation treatment and moderate neutropenia (1-14 days) (Table 3). HEPA filters did not appear to reduce the rates of IFIs in non-neutropenic patients or in patients with >14 days of neutropenia, patients undergoing induction treatment, or patients with either acute myeloid leukemia or non-acute leukemia (multiple myeloma, solid tumors, lymphoma, etc.) (Table 3).

We also evaluated the patients’ hospital bills per group. The total cost of the HEPA filters, including costs of installment and service over the 6-month intervention period, was 50,975 Turkish lira (TL; equivalent to 29,809 US$ or 21,328 €) [15]. We found that all costs as given in dollars and euros per patient were decreased after HEPA filter installation, but costs as expressed in TL were not significantly different between these groups (Table 4).

## DISCUSSION

Hospital construction is a significant source of serious hospital-acquired infections due to aspergillosis, with nosocomial aspergillosis outbreaks occurring primarily among neutropenic patients [16]. The period before the construction, when the HEPA filters had not yet been installed, was winter and spring, while the installed HEPA filters were used in summer and autumn. The use of HEPA filters was associated with a lack of increase in IFI rates despite both the construction and the summer months [17]. We conclude that HEPA filter installation in hematology wards is a safe option to prevent IFIs during construction. The use of HEPA filters most likely prevented the rates of infection-related mortality from increasing in patients treated during construction.

Clinically documented infections originate from either IFIs or bacterial infections. The pulmonary system is the origin of most infections, but other systems may also be involved [18,19]. The most common bacterial agents observed in our study were similar to those reported to be most common in the literature [20]. HEPA filters prevented both IFIs and bacterial infections [21]. In our study, the rates of clinically documented pneumonia were also reduced, which may explain why the use of HEPA filters decreased the rates of clinically documented infections. The literature contains few reports about the effects of HEPA filters on patients with hematological malignancies and either clinically documented infections or clinically documented pneumonia; to our knowledge, our study is the first to report this clinical finding.

During construction, the IFI rates did not increase in the subsets of patients who were at higher risk of IFIs, including those with acute myeloid leukemia, those undergoing remission-induction therapy, and/or those with neutropenia that lasted >14 days. This outcome was most likely due to the ability of HEPA filters to prevent IFIs. However, HEPA filters appeared to be most effective in preventing infections in patients with moderate duration of neutropenia, patients with acute lymphoid leukemia, and patients undergoing consolidation therapy. These groups are reported in the literature to have lower rates of IFIs [22]. This might result from a balance between the protective effects of the HEPA filters and the deleterious effects of neutropenia duration on developing IFIs. To our knowledge, this finding has not yet been reported in the literature. In multi-center studies, the effect of HEPA filters in preventing infections may be a confounding variable, and HEPA filter effects should be taken into account.

HEPA filters can reduce the exposure to Aspergillus from unfiltered air and contaminated dust by reducing the number of Aspergillus organisms in the air [23]. Aspergillus has been cultured from numerous hospital sources including horizontal surfaces, food, water supplies, and ventilation systems [24]. HEPA filters may not completely prevent IFI in high-risk patients [16]. As a result, antifungal prophylaxis should be considered as another preventive option in high-risk patient groups [6,25,26].

The effect of season on IFI is controversial. It has been reported that aspergillosis infections are most commonly seen in the summer [7]. However, one study found no seasonal effect on the rate of IFIs [27]. In our study, we were not able to evaluate seasonal effects on the incidence of IFIs because of the study design. However, Bénet et al. reported that the incidence of IFIs in hematological patients during the summer months in the absence of HEPA filters was 13.2% (9/68) [28]. We observed that the IFI incidence during the winter months in the absence of HEPA filters was 9.5% (20/210). Our study population and that of Bénet et al. [28] were similar. Thus, we compared the findings of our study with those of Bénet et al. [28] to evaluate seasonal effect on the rate of IFIs. There was no significant difference between the summer and winter IFI rates in these studies (p=0.4). In other words, the protective effects of HEPA filters against infections were independent of season.

The duration of hospitalization was longer before the installation of HEPA filters than after installation. Lower incidences of infection in the intervention group during construction may have led to shorter hospital stays.

Adal et al. reported that HEPA filters may be cost-effective [29]. We did not evaluate the cost-effectiveness of HEPA filters in our patients. However, we found that HEPA filter installation lowered all costs per patient in euro and dollar currencies, although costs as expressed in TL were not significantly different between these groups, probably due to the changes in exchange rates. Thus, we propose that HEPA filters may be a cost-effective option for preventing infections in hematology patients, especially when construction is taking place nearby.

Our study had several limitations, including its retrospective nature, a small sample size, the fact that it was conducted at a single center, and the lack of cost-benefit analysis. In addition, our confirmed IFIs rates were low, because they were not evaluated by pathology.

Some studies found hypoalbuminemia to be a risk factor for Aspergillus infections [30,31]. Therefore, we evaluated minimum albumin levels in patients treated in HEPA and non-HEPA rooms. However, we did not observe any differences in albumin levels between these 2 patient groups.

## CONCLUSION

In conclusion, after the implementation of infection control measures during construction, we found that keeping immunocompromised patients in single-bed rooms with air filtration through a HEPA system could significantly reduce IFIs in low-risk patient groups. However, additional protective measurements such as antifungal prophylaxis are required to reduce the rate of infection in high-risk patient groups.

## Ethics

Ethics Committee Approval: Retrospective study, Informed Consent: It was taken.

## Figures and Tables

**Table 1:**
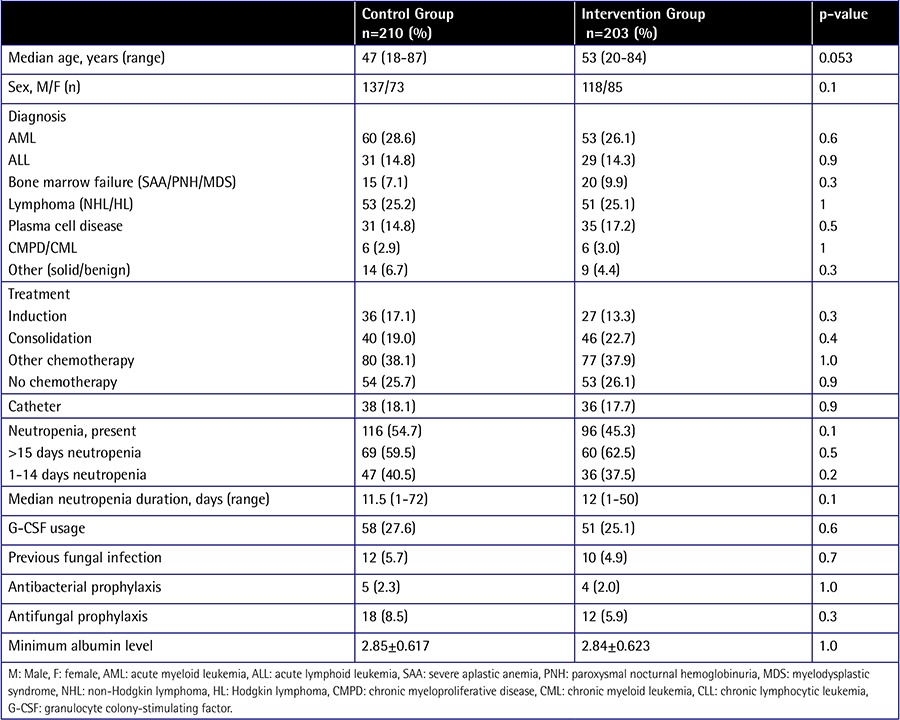
Patient characteristics.

**Table 2:**
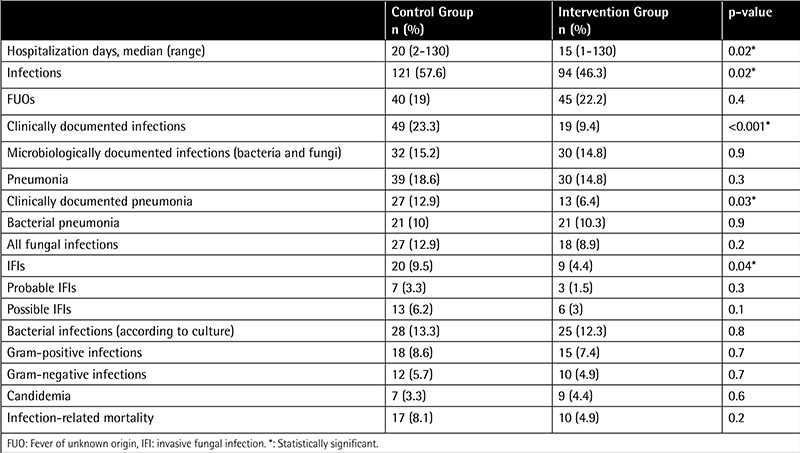
The effect of high-efficiency particulate absorption filters on infection rates.

**Table 3:**
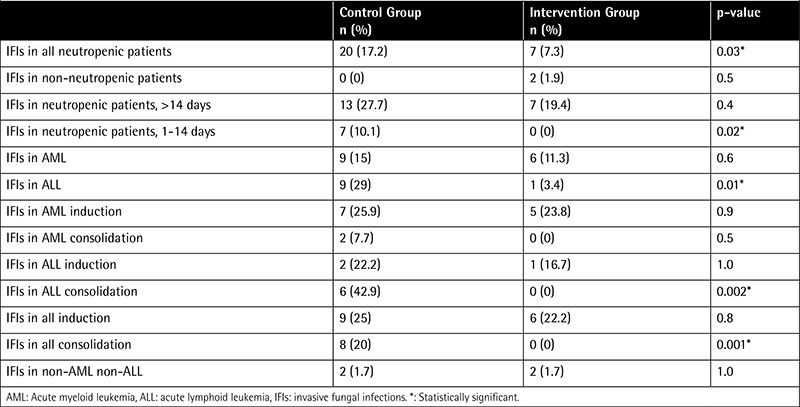
The effect of high-efficiency particulate absorption filters on invasive fungal infections.

**Table 4:**
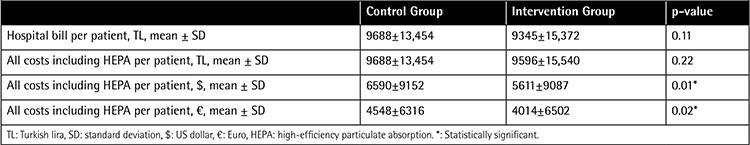
Financial analysis of the patients.
